# The relationship between nurses' risk assessment and management, fear perception, and mental wellbeing during the COVID-19 pandemic in Saudi Arabia

**DOI:** 10.3389/fpubh.2022.992466

**Published:** 2022-11-10

**Authors:** Reem N. Al-Dossary, Sana AlMahmoud, Maram Ahmed Banakhar, Majed Alamri, Hamdan Albaqawi, Khaled Al Hosis, Mohammed S. Aljohani, Bader Alrasheadi, Rawaih Falatah, Noura Almadani, Khalid Aljohani, Jalal Alharbi, Joseph U. Almazan

**Affiliations:** ^1^Nursing Education Department, Nursing College, Imam Abdulrahman Bin Faisal University, Dammam, Saudi Arabia; ^2^Public Health Nursing Department, Faculty of Nursing, King Abdul-Aziz University, Jeddah, Saudi Arabia; ^3^Nursing Department, College of Applied Medical Sciences, University of Hafr Al Batin, Hafar Al Batin, Saudi Arabia; ^4^College of Nursing, University of Hail, Hail, Saudi Arabia; ^5^Department of Nursing Education, Nursing College, Qassim University, Buraydah, Saudi Arabia; ^6^Medical and Surgical Department, Nursing College, Taibah University, Medina, Saudi Arabia; ^7^Nursing Department, College of Applied Medical Sciences, Majmaah University, Al Majma'ah, Saudi Arabia; ^8^Nursing Administration and Education Department, College of Nursing, King Saud University, Riyadh, Saudi Arabia; ^9^Community Health Nursing Department, College of Nursing, Princess Nourah Bint Abdulrahman University, Riyadh, Saudi Arabia; ^10^Community Health Nursing Department, Nursing College, Taibah University, Medina, Saudi Arabia; ^11^Nursing Department, College of Applied Medical Sciences, University of Hafr Albatin, Hafar Al Batin, Saudi Arabia; ^12^Medicine Department, Nazarbayev University School of Medicine, Nazarbayev University, Astana, Kazakhstan

**Keywords:** COVID-19, fear perception, risk assessment and management, mental wellbeing, pandemic

## Abstract

During this pandemic, it is crucial to implement early interventions to help nurses manage their mental wellbeing by providing them with information regarding coping skills, preventive risk assessment approaches (such as hospital preparedness and rapid risk assessment), and the ability to respond. This study evaluated the effect of fear and risk assessment management on nurses' mental wellbeing during the COVID-19 pandemic in Saudi Arabia. A total of 507 nurses who worked in tertiary public hospitals were asked to take a descriptive design survey. Three survey scales were used to assess the survey: the Risk Assessment Scale, the Fear of COVID-19 Scale, and the Warwick-Edinburgh Mental Wellbeing Scale. Independent *t*-tests and a one-way ANOVA were used to examine the association between fear of COVID-19 and nurses' demographic characteristics on their mental wellbeing. A multiple regression analysis was performed to examine the predictors associated with mental wellbeing. Findings revealed that almost half of the participants showed moderate positive mental wellbeing, 49.7%, while only 14% had low levels of fear on the Warwick-Edinburgh Mental *Well* being Scale. Most of the respondents had low levels of fear on the Fear of COVID-19 Scale, 45%, while only 15% had high levels of fear on the scale. Then, some demographic variables, such as “age,” “nationality,” “total years of experience in the current hospital,” and “region you work at” had statistically significant differences with *p* < 0.5. Meanwhile, risk assessment is also associated with mental wellbeing scores. All items on the Fear of COVID-19 Scale showed no significant difference with a *P* > 0.05. In conclusion, most nurses providing direct patient care to a patient with COVID-19 emphasized the importance of wearing PPE and performing hand hygiene before and after any clean or aseptic procedure. Meanwhile, although almost all nurses were vaccinated, they were still afraid of a COVID-19 infection. Additionally, the results reported that the older the nurses are, the better their mental wellbeing scores. Non-Saudi nurses had higher perceived mental wellbeing scores than Saudi nurses, and different working environments corresponded to different mental wellbeing scores. Finally, nurses' risk assessment was associated with mental wellbeing scores.

## Introduction

The coronavirus disease 2019 (COVID-19) pandemic has unsympathetically put the health of healthcare professionals (HCPs) and the public at unprecedented health risks. This has prompted immediate action from medical facilities around the world. Two years after the COVID-19 outbreak, as of 2 May 2022, data showed that 66.2% of the world population has already received the vaccine, which is more than 5.08 billion people around the world (1). According to the World Health Organization's (1) epidemiological update regarding COVID-19, there was a 16% decline in newly reported cases from 28 March 2022 to 3 April 2022, and both new weekly cases and deaths have been declining globally (1). However, although the data speak positively on a macro-level scale, individuals are nevertheless confronted with challenges at the micro level. For instance, in some countries, HCPs were assigned as one of the priority healthcare groups for vaccination (2, 3). A recent Centers for Disease Control and Prevention study published in 2021 (4) reported that vaccination has a greater protection rate than those previously infected with COVID-19. There was high anticipation from the HCPs for these vaccines. Nurses make up one of the largest groups of HCPs and have the most direct patient contact, so vaccination is crucial for them because it reduces their risk of infection (5).

By the end of 2021, over 50% of healthcare professionals (including nurses) worldwide were neither fully vaccinated nor had no plans to do so (6). For instance, vaccination hesitancy has been one of the determinants of under-vaccination in some nurses (7). According to Reses et al. (3), the reason for the high hesitancy status of getting vaccinated can be attributed to people's political views and a lack of confidence and trust in vaccine manufacturers. Moreover, people were hesitant to get the COVID-19 vaccination due to their concerns regarding its side effects, which stemmed from the vaccine's quick development and the conspiracy theory that it is employed for population control (8–10). The rapid pace of vaccine development and rollout may have caused doubts regarding vaccine safety. Meanwhile, another study found the cause of greater reluctance to vaccination to be the vaccine development process and a lack of knowledge about the vaccine's long-term effects (11).

Similar to vaccination reluctance, the physical and mental wellbeing of HCPs, particularly frontline nurses, is becoming a significant concern over the pandemic's larger impact due to the unknown number of COVID-19-related excess deaths among HCPs (12). COVID-19 also impacted the workforce in the healthcare sector. Undoubtedly, nurses were most severely affected by the pandemic, as they faced multiple hazards that affected their physical as well as mental wellbeing (13). In addition, Al-Dossary et al. (14) explained that nurses experienced moderate to severe symptoms of burnout, distress, anxiety, fear, and trauma during the COVID-19 pandemic. Another study from Bangladesh about the challenges faced by HCPs reported that nurses experienced greater workloads, scarce incentives, psychological distress, personal protective equipment (PPE) shortages, and social exclusion/stigmatization (15).

In addition, the disproportionate impact of the COVID-19 pandemic on hospitals has resulted in nursing staff shortages and increased workload (16), which could lead to stress and exhaustion for nurses. This heavy workload negatively affects patient safety, causes greater stress, reduces job satisfaction, and increases occupational burnout. Increased demands on nurses' time and energy lead to a decline in their quality of life (17) and can have negative consequences for patient care in the form of staffing shortages and high turnover rates.

When the pandemic hit Saudi Arabia, it exacerbated an already problematic situation for nursing professionals. A recent study revealed that nurses dreaded the disease for a variety of reasons, including the abrupt increase in COVID-19-related morbidity and mortality in the country (18, 19). Moreover, nurses reported being directly involved in the treatment of patients diagnosed positive for COVID-19. Thus, they were apprehensive about contracting the infection and passing it on to their families. Furthermore, nurses, like any human being, fear the unknown. During the first few months of the pandemic, the lack of adequate disease knowledge and no specific treatment caused the nurses to be exposed to transmission risk and emotional distress (14, 20). Nurses' anxiety about COVID-19 may have been influenced by any number of factors, many of which have been the subject of prior research. As reported by recent studies, assessing the nurses' fear and mental wellbeing during the outbreak of COVID-19 has been seen as a crucial matter. For example, the investigation by Albaqawi et al. (21) regarding the nurses' risk for COVID-19 infection revealed that nurses who do not practice proper hand hygiene after physical contact with patients with COVID-19 and who do not replace PPE are at a higher risk of contracting the infection. Additionally, nurses' inadequate understanding of COVID-19, inadequate PPE, lack of access to tests, and the presence of psychological stress were the major factors for infection among healthcare professionals (22, 23), thus highlighting the importance of COVID-19 preparedness in managing its risks to HCPs. A study conducted in the Najran Region, KSA, revealed that nurses are well-versed in preventive measures and reported attending orientation about the COVID-19 guidelines (24). This is probably why early interventions help the nurses manage their mental wellbeing by providing them with information regarding coping skills and, at the same time, the preventive risk assessment approach (e.g., hospital preparedness and rapid risk assessment) and the ability to respond during this pandemic is greatly important (25).

Previous studies examined how they manage the emotional distress brought on by the pandemic, and it was revealed that nurses' use of COVID-19 protective measures such as PPE, psychological and management support, avoidance strategies, faith-based practices, and social support from their families during the COVID-19 outbreak improved nurses' coping response (26, 27).

A study identified informational support, such as training and clear preventative programs, as one of the factors that may treat mental health issues. It has been demonstrated that instrumental support is particularly effective in providing adequate PPE and other protection protocols. Emotional and psychological support, such as counseling and therapy for nurses experiencing emotional distress, and organizational support, such as manpower allocation and redistribution of workloads and working hours, were themes that were revealed to be helpful not only to nurses but also to policymakers in each healthcare institution (28). The strain on mental health can cause dysfunction at work and other disorders that could be more serious if left untreated. Actions such as risk management and risk reduction for all staff can be taken to mitigate this risk.

The interventions, such as spreading awareness through public health education, helped the HCPs manage their fear and psychological wellbeing throughout the pandemic. A study has shown that public health education decreased the fear of an individual toward COVID-19 since the awareness of the disease helped nursing professionals understand that preventive measures can work effectively (29). Time management training for the nurses was also found to be helpful for their mental health during the COVID-19 pandemic. Sun (30) conducted a 16-week intervention for the nurses, and the results suggested that the training significantly increased the mental health level of the nurses. Mental health interventions, such as emotional management, were given to the HCPs and could enhance the ability of the nurses to regulate their emotions and help them prevent mental disorders (31). Previous studies proved that interventions had positive effects on nurses. Ali et al. (22) discussed that the number of HCPs getting infected with COVID-19 is increasing and that one of the major risk factors is the lack of understanding of the disease. Thus, they suggested that governing bodies should focus on providing education and training to increase the readiness of their staff. The same suggestion was also made in the study by d'Ettorre et al. (32), where they advised a need for interventions to protect against the mental health effects brought by the pandemic and management strategies for posttraumatic stress symptoms. A risk assessment strategy can successfully decrease the occurrence of risk events in nursing practice (33).

Therefore, it is also safe to presume that the risk assessment and management of the HCPs could also influence their undue fear and mental wellbeing during the COVID-19 pandemic. While there are studies investigating the risk assessment of the HCPs, particularly the nurses, to the best of the researchers' ability, there has been no study conducted in Saudi Arabia exploring the direct relationship of risk assessment management to the fear and mental wellbeing of nurses. Mental wellbeing is an integral and essential component of nurses' health, impacting patient safety and the quality of care provided. Evaluating the effect of risk assessment management on nurses' mental wellbeing and fear is significant, particularly in the nursing profession, since it helps healthcare institution policymakers better combat nurses' fear and mental wellbeing. In this study, the information on how healthcare professionals fear COVID-19, which, in turn, will support further designing of appropriate programs to take care of their fear and mental wellbeing, could also ensure that they deliver quality care, thereby maintaining the safety of the patients. Maintaining the good mental health conditions of the HCWs would mean that they are fit to perform the task at their optimum level. When the nurses work at their best, they become efficient. In addition, better mental health increases HCP satisfaction and decreases voluntary turnover, which is a cost-saving move for hospital administration due to the reduction or elimination of a number of charges and losses that would otherwise be incurred (34).

Finally, the result could provide baseline information on risk assessment on reducing the transmission rate of COVID-19 and promoting positive mental wellbeing. Having established the impact of COVID-19 on the fear and mental health of nurses providing direct patient care in Saudi Arabia, this study is crucial for advancement not only in nursing research but also for the nursing profession in Saudi Arabia. This study evaluated the effect of risk assessment management on nurses' mental wellbeing and fear during the COVID-19 pandemic in Saudi Arabia. It is currently relevant that a new study will be conducted, especially since the COVID-19 pandemic is ongoing. The mere presence of the pandemic simply means that the challenges for nurses remain. Thus, the study seeks to determine how HCPs manage the risks posed by the pandemic, as well as its effect on nurses' fear and mental health.

## Materials and methods

This study is cross-sectional in design. We used online survey questionnaires to gather data from the selected tertiary public hospitals in Saudi Arabia. The nurses were eligible to become participants in the study if they had a license and had at least 1 year of hospital experience. We are presently working in a Saudi Arabian public or private hospital that has provided care to patients with COVID-19 and gave consent to participate in the study.

### Data collection

This study was approved by the Ministry of Health Ethical Review Board (Institutional Review Board–Hafr Albatin committee (KACST No. H-05-FT-083). Each survey questionnaire was accompanied by a cover letter describing the study's goal, the participant's right to decline participation, and the fact that participation implies consent. Confidentiality was ascertained as the survey questionnaire did not ask for any data that could expose the identity of the participants. After ethical approval was obtained, the researcher sent a copy of the survey questionnaire to each department in the selected tertiary hospitals for approval. Permission to use the tools was sent to each author of the tool, and approval letters from the authors were received. After the participants were identified, the questionnaires were distributed. Participants were asked to complete the questionnaire during their respective free time to avoid disturbing their work. Data were collected from February to May 2022.

### Sample size

The sample size was decided using the online Sample Size Calculator (https://www.calculator.net/sample-size-calculator.html). This means 385 or more surveys were needed to have a 95% confidence level in which the real value was within ±5% of the surveyed value. In this study, a total of 507 nurses answered the survey, which is above the minimum sample requirements.

### Survey questionnaire

The tool is a four-part questionnaire:

#### Demographic data

This section describes respondents' demographic and work-related characteristics.

#### Risk assessment scale

This scale is a modified version of the WHO's COVID-19 Virus Exposure Risk Assessment Form for HCPs. This tool contains three subparts that assess the risk category of nurses after exposure to a patient with COVID-19 (25). The first part contains the five items on nurses' intervention conducted on patients with COVID-19 in clinical settings. The second part contains the 7-item adherence to IPC procedures during healthcare interactions. Finally, the third part contains 5-item questions regarding the COVID-19 vaccine. In this study, this tool's reliability was 0.91 (Cronbach's alpha).

#### Warwick–Edinburgh mental wellbeing scale

The tool was composed of 14 items that assessed the mental state of the nurses during the COVID-19 outbreak (35). The participants' responses were measured on a 5-point scale (1 = none of the time to 5 = all of the time), with higher mean scores meaning greater positive mental wellbeing. The minimum score on the scale is 14, while the maximum score that can be attained is 70. A higher score means improved mental wellbeing. Several studies performed in various countries on the psychometric properties of the tools found that they have acceptable reliability and validity for assessing an individual's mental wellbeing (13, 36, 37). This study has a reliability of 0.95 (Cronbach's alpha).

#### Fear of COVID-19 scale

The tool measures anxiety and fear of COVID-19 (38). This tool comprises a 7-item self-administered questionnaire and has been psychometrically validated in several countries (39–42). Nurses indicated their level of agreement with the statements using a five-item Likert-type scale. Answers included “strongly disagree,” “disagree,” “neutral,” “agree,” and “strongly agree.” The minimum score possible for each question was 1, and the maximum was 5. A total score could be calculated by adding up each item's score (ranging from 7 to 35). A higher overall score attained indicated severe fear of COVID-19. This study's computed Cronbach's alpha was 0.92, indicating high reliability.

### Data analysis

Demographic variables, risk assessment, fear of COVID-19, and mental wellbeing were frequently counted, and percentages, mean scores, and standard deviations were calculated. The Shapiro–Wilk test was applied to check the normality or distribution of data. It found that if the value of the Shapiro–Wilk and Kolmogorov-Smirnova tests is >0.05, the data are normal. Pairwise deletion of cases was used with missing data. Independent *t*-tests and one-way ANOVA were used to examine the association between fear of COVID-19 and nurses' demographic characteristics on their mental wellbeing. Multiple regression analysis examined the predictors associated with mental wellbeing. The dependent variable used is the Warwick–Edinburgh Mental Well-Being Scale, and the independent variables are the nurses' demographic characteristics and fear of COVID-19. A *p*-value of 0.05 is considered significant.

## Results

Table 1 shows that the total number of participants was equal to 507. Most of the participants were women (86.2%), and the age of most participants ranged between 31 and 40 years (47.3%), while the participants aged 61 years and above had the lowest percentage (0.2%). More than half of the participants were married, with a percentage of 66.1%, while only 3.4% of them were separated. The participants' education level had 66.7% bachelor's degrees, while only 0.2% had doctorate degrees. The participants with non-Saudi nationality represented 57.8%, while those with Saudi nationality represented 42.2% of the participants. One-third of the participants (32.3%) had an experience of 1–5 years, and only 6.5% had an experience of 21 years or more. The central region (Riyadh, Qassim) represented the majority region where 46.7% of participants worked, followed by the Eastern Region (39.6%), the Northern Region (3.2%), the Southern Region (4.7%), and the Western Region (5.7%). Most of the participants were assigned to ICUs (15.4%), emergency departments (15.2%), and obstetric departments (15.6%), comprising almost half of the participants.

**Table 1:** Respondents' demographic data (*N* = 507).

**Demographic data**		**Frequency**	**Percent**
Age	20–30 years old	145	28.6
	31–40 years old	240	47.3
	41–50 years old	83	16.4
	51–60 years old	38	7.5
	61 years old and above	1	0.2
Gender	Male	70	13.8
	Female	437	86.2
Marital status	Married	335	66.1
	Separated	17	3.4
	Signal	149	29.4
	Widow	6	1.2
Educational attainment	Diploma	119	23.5
	Bachelor	338	66.7
	Master	49	9.7
	PhD	1	0.2
Nationality (Saudi and Non-Saudi only)	Saudi	214	42.2
	Non-Saudi	293	57.8
Total years of experience in the current hospital	1–5 years	164	32.3
	6–10 years	131	25.8
	11–15 years	105	20.7
	16–20 years	74	14.6
	21 years and more	33	6.5
Region	Central region (Riyadh, Qassim)	237	46.7
	Eastern region (Dammam, Jubail, Hassa, & others)	201	39.6
	Northern region (Hail, Aljouf, Tabouk, & Arar)	16	3.2
	Southern region (Assir, Jazan, Najran, Baha)	24	4.7
	Western region (Makkah, Jeddah, Taif, & Madinah)	29	5.7
Area of practice	Artificial kidney unit	31	6.1
	Emergency department	77	15.2
	Medical department	45	8.9
	Intensive care unit	78	15.4
	Nursing administration	49	9.7
	Operating room	24	4.7
	Outpatient department	42	8.3
	Pediatric department	57	11.2
	Surgical department	25	4.9
	Obstetric department	79	15.6

The perceived risk assessment of the respondents is shown in Table 2. The first part of the risk assessment tool consists of five questions reflecting the following: 52.3% of the participants lived with their families at the pandemic's peak. In contrast, the remaining lived in an isolated room, where 83.2% of participants provided direct care to a confirmed COVID-19 patient, 81.5% had face-to-face contact within 1 m with a patient confirmed with COVID-19 in a health care facility, 72.2% were present when any aerosol-generating procedures were performed on the patient, and 78.1% had direct contact with the environment where the patient was confirmed for COVID-19. The second part of the risk assessment tool consisted of seven questions, which reflects the following: 94.28% of the participants wore PPE (62.13% medical masks, 32.94% face shields or goggles, and 4.93% single gloves), 86.19% followed the protocols by removing and replacing their PPE, 88.17% “always practice hand hygiene before and after touching the COVID-19 patient,” 89.3% “always practice hand hygiene before and after any clean or aseptic procedure was performed,” 92.70% “always practice hand hygiene after exposure to body fluid, 84.62% perform hand hygiene after touching the patient's surroundings,” and 65.09% of them “always have high touch surfaces decontaminated frequently.” The third part of the risk assessment tool consisted of five questions, which reflects the following: 98.42% of the participant were vaccinated, 79.09% of them had experienced side effects related to the vaccine, 53.65% had negative PCR test, 75.54% had adequate personal protective equipment (PPE) when working with patients infected with COVID-19, and 63.12% of the participants were afraid of becoming infected while working with patients tested positive for COVID-19 (Table 2).

**Table 2:** Perceived risk assessment of the respondents (*N* = 507).

**Risk assessment**		**Parameters**	**Frequency**	**Percent**
Part 1	1. At the peak of the pandemic where were you living?	Isolated from family	242	47.7
		Living with family	265	52.3
	2. Did you provide direct care to a confirmed COVID-19 patient?	No	85	16.8
		Yes	422	83.2
	3. Did you have face to face contact within 1 m with a confirmed COVID-19 patient in a health care facility	No	94	18.5
		Yes	413	81.5
	4. Were you present when any aerosol-generating procedures were performed on the patient?	No	141	27.8
		Yes	366	72.2
	5. Did you have direct contact with the environment where the confirmed COVID-19 patient was cared for? E.g., bed, linen, medical equipment, bathroom etc	No	111	21.9
		Yes	396	78.1
Part 2	6. During a health care interaction with a COVID-19 patient, did you wear personal protective equipment (PPE)?	No	29	5.72
		Yes	478	94.28
	What PPE?	Single gloves	25	4.93
		Medical mask	315	62.13
		Face shields or goggles	167	32.94
	7. During a health care interaction with the COVID-19 patient, did you remove and replace your PPE according to protocol (e.g., when medical mask became wet, disposed the wet PPE in the waste bin, performed hand hygiene, etc)	Most of the time (50% or more but not 100%)	70	13.81
		Always, as recommended	437	86.19
	8. During a health care interaction with the COVID-19 patient, did you perform hand hygiene before and after touching the COVID-19 patient (whether or not you were wearing gloves)?	Never	2	0.39
		Sometimes	5	0.99
		Most of the time	53	10.45
		Always	447	88.17
	9. During a health care interaction with the COVID-19 patient, did you perform hand hygiene before and after any clean or aseptic procedure was performed (e.g., while inserting a peripheral vascular catheter, urinary catheter, intubation, etc)?	Never	5	1.0
		Rarely	2	0.4
		Sometimes	7	1.4
		Most of the time	40	7.9
		Always	453	89.3
	10. During a health care interaction with the COVID-19 patient, did you perform hand hygiene after exposure to body fluid?	Never	4	0.79
		Sometimes	5	0.99
		Most of the time	28	5.52
		Always	470	92.70
	11. During a health care interaction with the COVID-19 patient, did you perform hand hygiene after touching the patient's surroundings (bed, door handle, etc.), regardless of whether you were wearing gloves?	Never	5	0.99
		Rarely	2	0.39
		Sometimes	9	1.78
		Most of the time	62	12.23
		Always	429	84.62
	12. During a health care interaction with the COVID-19 patient, were high touch surfaces decontaminated frequently (at least three times daily)?	Never	8	1.58
		Rarely	10	1.97
		Sometimes	32	6.31
		Most of the time	127	25.05
		Always	330	65.09
Part 3	13. Are you vaccinated?	No	8	1.58
		Yes	499	98.42
	14. Did you experience any side effect related to the vaccine:	No	106	20.91
		Yes	401	79.09
	15. Have you been confirmed to have a positive (PCR) test of (COVID-19)?	No	272	53.65
		Yes	235	46.35
	16. At the peak of pandemic, were there adequate personal protective equipment PPE during working with infected patients of (COVID-19)?	No	124	24.46
		Yes	383	75.54
	17. Were you afraid of being infected with (COVID-19) while working with a positive patient?	No	187	36.88
		Yes	320	63.12

Table 3 shows the respondent's perceived mental wellbeing using the WEMWBS.

**Table 3:** Respondent's perceived mental wellbeing using the WEMWBS.

**No**	**Statement**	***N* %**	**None**	**Rarely**	**Sometimes**	**Often**	**All the time**	**Mean ±SD**
1	I've been feeling optimistic about the future	*N*	43	71	189	108	96	3.28 ± 1.17
		%	8.5	14	37.3	21.3	18.9	
2	I've been feeling useful	*N*	27	67.0	160	125	128	3.51 ± 1.16
		%	5.3	13.2	31.6	24.7	25.2	
3	I've been feeling relaxed	*N*	57	128	203.0	82.0	37	2.83 ± 1.06
		%	11.2	25.2	40	16.2	7.3	
4	I've been feeling interested in other people	*N*	33	100	216	104.	54	3.09 ± 1.04
		%	6.5	19.7	42.6	20.5	10.7	
5	I've had energy to spare	*N*	27	105	206.0	114.0	55	3.13 ± 1.03
		%	5.3	20.7	40.6	22.5	10.8	
6	I've been dealing with problems well	*N*	17.0	73	200.0	147.0	70.0	3.36 ± 1
		%	3.4	14.4	39.4	29.0	13.8	
7	I've been thinking clearly	*N*	13	71	177.0	148.0	98	3.49 ± 1.04
		%	2.6	14	34.9	29.2	19.3	
8	I've been feeling good about myself	*N*	23	73	161.0	138.0	112.0	3.48 ± 1.12
		%	4.5	14.4	31.8	27.2	22.1	
9	I've been feeling close to other people	*N*	26	97	174.0	143.0	67	3.25 ± 1.07
		%	5.1	19.1	34.3	28.2	13.2	
10	I've been feeling confident	*N*	27	64	164.0	148.0	104	3.47 ± 1.11
		%	5.3	12.6	32.3	29.2	20.5	
11	I've been able to make up my own mind about things	*N*	23	73.0	170.0	138	103	3.44 ± 1.1
		%	4.5	14.4	33.5	27.2	20.3	
12	I've been feeling loved	*N*	24	77	172	134	100	3.44 ± 1.1
		%	4.7	15.2	33.9	26.4	19.7	
13	I've been interested in new things	*N*	29	76	174.0	120.0	108	3.4 ± 1.15
		%	5.7	15	34.3	23.7	21.3	
14	I've been feeling cheerful	*N*	27	86	175	129	90	3.33 ± 1.11
		%	5.3	17	34.5	25.4	17.8	

Items 1, 3, 4, 5, 6, 9, and 14 had a moderate mean ranging between 3.09 and 3.36, while items 2, 7, 8, 10, 11, 12, 13, and 14 had a high mean ranging between 3.4–3.51. The WEMWBS was converted into three categories, which are low, moderate, and high. Most of the participants had moderate levels, with a percentage of 49.7%, while only 14% had low levels of mental scale. This result implies a perceived moderate positive mental wellbeing among nurses.

The association between respondents' demographic profile and perceived risk assessment about their mental wellbeing scores is shown in Table 4. Items 1, 2, 4, and 5 have results of “neutral” with a mean range between 2.76–2.95, while items 3, 6, and 7 have results of “disagree” with a mean range between 2.26–2.32. The Fear of the COVID-19 Scale was converted into three categories, which are low, moderate, and high levels. Most of the respondents had a low level of fear, 45%, while only 15% had high levels of fear on the scale. This implies that respondents had a low fear of COVID-19.

**Table 4:** Respondents scores in fear of COVID-19.

**No**	**Question**	***N* %**	**Strongly disagree**	**Disagree**	**Neutral**	**Agree**	**Strongly agree**	**Mean ±SD**
1	I am most afraid of Corona	*N*	84.00	88	164.00	112	59	2.95 ± 1.23
		%	16.57	17.36	32.35	22.09	11.64	
2	It makes me uncomfortable to think about Corona	*N*	71	127	141	129	39	2.88 ± 1.17
		%	14	25.05	27.81	25.44	7.69	
3	My hands become clammy when I think about Corona	*N*	137	166	128	55	21	2.32 ± 1.11
		%	27.02	32.74	25.25	10.85	4.14	
4	I am afraid of losing my life because of Corona	*N*	100	119	129	100	59	2.8 ± 1.28
		%	19.72	23.47	25.44	19.72	11.64	
5	When I watch news and stories about Corona on social media. I become nervous or anxious	*N*	88	129	146	106	38	2.76 ± 1.18
		%	17.36	25.44	28.80	20.91	7.50	
6	I cannot sleep because I'm worrying about getting Corona	*N*	164	153	105	64	21	2.26 ± 1.16
		%	32.35	30.18	20.71	12.62	4.14	
7	My heart races or palpitates when I think about getting Corona	*N*	166	146	114	58	23	2.26 ± 1.16
		%	32.74	28.80	22.49	11.44	4.54	

Table 5 shows the relationship between respondents' demographic profile and their perceived risk assessment of their mental wellbeing scores. As shown in the Table 5, “age,” “nationality,” “total years of experience in the current hospital,” and “region you work at” had statistically significant differences with P-values less than 0.5 as follows 0.048, 0.008, 0.036, and 0.004, respectively. In contrast, “gender,” “marital status,” “educational attainment,” and “area of practice” had no significant difference with a *P* > 0.05. Meanwhile, risk assessment is also associated with mental wellbeing scores with a statistical significance difference with a *P* < 0.5.

**Table 5:** Association between respondents' demographic profile and perceived risk assessment in relation to their mental wellbeing scores.

**Demographic characteristics**		**Warwick Edinburgh mental wellbeing scale**				**Statistical test**	***P*-Value**
		**Low level**	**Moderate level**	**High level**	**Mean ±SD**		
Gender	Male	9 (13%)	37 (53%)	24 (34%)	3.34 ± 1.01	*t* = 0.254	0.799
	Female	62 (14%)	215 (49%)	160 (37%)	3.32 ± 0.87		
Age	20–30 years old	17 (12%)	82 (57%)	46 (32%)	3.28 ± 0.85	*F* = 3.045	0.048*
	31–40 years old	38 (16%)	117 (49%)	85 (35%)	3.3 ± 0.9		
	41–50 years old	11 (13%)	39 (47%)	33 (40%)	3.38 ± 0.91		
	51–60 years old	4 (11%)	14 (37%)	20 (53%)	3.54 ± 0.95		
	61 years old and above	1 (100%)	0 (0%)	0 (0%)	–		
Marital status	Married	44 (13%)	166 (50%)	125 (37%)	3.34 ± 0.89	*F* = 0.377	0.686
	Separated	4 (24%)	7 (41%)	6 (35%)	3.13 ± 0.99		
	Single	21 (14%)	77 (52%)	51 (34%)	3.31 ± 0.88		
	Widow	2 (33%)	2 (33%)	2 (33%)	3 ± 0.96		
Educational attainment	Diploma	25 (21%)	58 (49%)	36 (30%)	3.1 ± 1.05	*F* = 1.042	0.354
	Bachelor	36 (11%)	172 (51%)	130 (38%)	3.4 ± 0.81		
	Master	10 (20%)	21 (43%)	18 (37%)	3.28 ± 0.97		
	PhD	0 (0%)	1 (100%)	0 (0%)	–		
Nationality	Saudi	47 (22%)	109 (51%)	58 (27%)	3.12 ± 0.97	*F* = 4.868	0.008*
	Non-Saudi	40 (7.89)	223 (57.59)	175 (34.52)	4.17 ± 0.93		
Total years of experience in the current hospital	1–5 years	18 (11%)	91 (55%)	55 (34%)	3.31 ± 0.79	*F* = 3.340	0.036*
	6–10 years	17 (13%)	68 (52%)	46 (35%)	3.32 ± 0.93		
	11–15 years	18 (17%)	48 (46%)	39 (37%)	3.32 ± 0.9		
	16–20 years	13 (18%)	34 (46%)	27 (36%)	3.22 ± 0.96		
	21 years and more	5 (15%)	11 (33%)	17 (52%)	3.54 ± 1.07		
Region, you work at	Central region (Riyadh, Qassim)	26 (11%)	111 (47%)	100 (42%)	3.43 ± 0.85	*F* = 5.465	0.004*
	Eastern region (Dammam, Jubail, Hassa, & others)	27 (13%)	112 (56%)	62 (31%)	3.24 ± 0.88		
	Northern region (Hail, Aljouf, Tabouk, & Arar)	3 (19%)	10 (63%)	3 (19%)	3.18 ± 0.75		
	Southern region (Assir, Jazan, Najran, Baha)	7 (29%)	6 (25%)	11 (46%)	3.3 ± 1.13		
	Western region (Makkah, Jeddah, Taif, & Madinah)	8 (28%)	13 (45%)	8 (28%)	3 ± 1.03		
Area of practice	Artificial kidney unit	4 (13%)	14 (45%)	13 (42%)	3.51 ± 0.91	*F* = 0.073	0.929
	Emergency department	13 (17%)	37 (48%)	27 (35%)	3.33 ± 0.95		
	Medical department	8 (18%)	25 (56%)	12 (27%)	3.08 ± 0.8		
	ICUs	7 (9%)	45 (58%)	26 (33%)	3.38 ± 0.78		
	Nursing administration	8 (16%)	19 (39%)	22 (45%)	3.38 ± 0.95		
	Operating room	3 (13%)	9 (38%)	12 (50%)	3.36 ± 0.93		
	Outpatient department	9 (21%)	20 (48%)	13 (31%)	3.15 ± 1		
	Pediatric department	3 (5%)	32 (56%)	22 (39%)	3.44 ± 0.79		
	Surgical department	4 (16%)	16 (64%)	5 (20%)	3.16 ± 1.02		
	Obstetric department	12 (15%)	35 (44%)	32 (41%)	3.31 ± 0.9		
**Risk assessment scale**							
Did you provide direct care to a confirmed COVID-19 patient?	No Yes	10 (12%) 61 (14%)	37 (44%) 215 (51%)	38 (45%) 146 (35%)	3.28 ± 0.89 3.3 ± 0.89	*t* = 1.139	0.255
Did you have face to face contact within 1 m with a confirmed COVID-19 patient in a health care facility	No Yes	13 (3%) 58 (14%)	38 (9%) 214 (51%)	43 (46%) 141 (34%)	3.47 ± 0.94 3.28 ± 0.88	*t* = 1.866	0.063
Were you present when any aerosol-generating procedures were performed on the patient?	No Yes	16 (4%) 55 (13%)	65 (15%) 187 (44%)	60 (43%) 124 (34%)	3.43 ± 0.9 3.28 ± 0.89	*t* = 1.73	0.084
Did you have direct contact with the environment where the confirmed COVID-19 patient was cared for? E.g., bed, linen, medical equipment, bathroom etc	No Yes	21 (5%) 50 (12%)	45 (11%) 207 (49%)	45 (41%) 139 (35%)	3.36 ± 1.01 3.31 ± 0.86	*t* = 0.518	0.605
During a health care interaction with the COVID-19 patient, did you remove and replace your PPE according to protocol (e.g., when medical mask became wet, disposed the wet PPE in the waste bin, performed hand hygiene, etc)	Most of the time (50% or more but not 100%) Always, as recommended	14 (3%) 57 (14%)	40 (9%) 212 (50%)	16 (23%) 168 (38%)	3.08 ± 0.89 3.36 ± 0.89	*t* = 2.369	0.017*
During a health care interaction with the COVID-19 patient, did you perform hand hygiene before and after touching the COVID-19 patient (whether or not you were wearing gloves)?	Never	2 (0%)	0 (0%)	0 (0%)	1.5 ± 0.71	*F* = 2.764	0.064
	Sometimes	1 (0%)	3 (1%)	1 (20%)	2.83 ± 1.27		
	Most of the time	7 (2%)	30 (7%)	16 (30%)	3.24 ± 0.82		
	Always	61 (14%)	219 (52%)	167 (37%)	3.34 ± 0.89		
During a health care interaction with the COVID-19 patient, did you perform hand hygiene before and after any clean or aseptic procedure was performed (e.g., while inserting a peripheral vascular catheter, urinary catheter, intubation, etc)?	Never Rarely Sometimes Most of the time Always	4 (1%) 0 (0%) 1 (0%) 5 (1%) 61 (14%)	1 (0%) 1 (0%) 4 (1%) 23 (5%) 223 (53%)	0 (0%) 1 (50%) 2 (29%) 12 (30%) 169 (37%)	2.07 ± 0.93 3.25 ± 1.06 3.29 ± 1.01 3.26 ± 0.79 3.34 ± 0.89	*F* = 4.209	0.015*
During a health care interaction with the COVID-19 patient, did you perform hand hygiene after exposure to body fluid?	Never	4 (1%)	0 (0%)	1 (20%)	2.24 ± 1.09	*F* = 2.174	0.115
	Rarely	1 (0%)	0 (0%)	1 (50%)	3.32 ± 2.37		
	Sometimes	1 (0%)	7 (2%)	1 (11%)	3.1 ± 0.62		
	Most of the time	7 (2%)	34 (8%)	21 (34%)	3.27 ± 0.77		
	Always	58 (14%)	211 (50%)	160 (37%)	3.34 ± 0.9		
During a health care interaction with the COVID-19 patient, did you perform hand hygiene after exposure to body fluid?	Never	4 (1%)	0 (0%)	1 (20%)	2.24 ± 1.09	*F* = 4.297	0.014*
	Rarely	1 (0%)	0 (0%)	1 (50%)	3.32 ± 2.37		
	Sometimes	1 (0%)	7 (2%)	1 (11%)	3.1 ± 0.62		
	Most of the time	7 (2%)	34 (8%)	21 (34%)	3.27 ± 0.77		
	Always	58 (14%)	211 (50%)	160 (37%)	3.34 ± 0.9		
During a health care interaction with the COVID-19 patient, were high touch surfaces decontaminated frequently (at least three times daily)?	Never	5 (1%)	1 (0%)	2 (25%)	2.48 ± 1.15	*F* = 2.106	0.123
	Rarely	4 (1%)	3 (1%)	3 (30%)	2.94 ± 1.12		
	Sometimes	2 (0%)	19 (5%)	11 (34%)	3.3 ± 0.77		
	Most of the time	12 (3%)	73 (17%)	42 (33%)	3.32 ± 0.73		
	Always	48 (11%)	156 (37%)	126 (38%)	3.35 ± 0.94		
Are you vaccinated?	No	5 (1%)	2 (0%)	1 (13%)	2.32 ± 0.93	*t* = 3.22	0.001*
	Yes	66 (16%)	250 (59%)	183 (37%)	3.34 ± 0.88		
Have you been confirmed to have a positive (PCR) test of (COVID-19)?	No	22 (5%)	144 (34%)	106 (39%)	3.44 ± 0.82	*t* = 3.181	0.002*
	Yes	49 (12%)	108 (26%)	78 (33%)	3.18 ± 0.95		
Were you afraid of being infected with (COVID-19) while working with a positive patient?	No Yes	21 (5%) 50 (12%)	92 (22%) 160 (38%)	74 (40%) 110 (34%)	3.38 ± 0.89 3.28 ± 0.89	*t* = 1.161	0.244

*Significant at 0.05 level.

Table 6 depicts the relationship between fear of COVID-19 and nurses' mental wellbeing scores. An ANOVA test was applied according to the data type. As shown in the table, all items on the Fear of COVID-19 Scale had a non-significant difference with a *P*-value of more than 0.05.

**Table 6:** Association between fear of COVID-19 to their mental wellbeing scores.

**Fear of COVID-19**		**Warwick Edinburgh mental wellbeing scale**				**Statistical test**	***P*-Value**
		**Low level**	**Moderate level**	**High level**	**Mean ±SD**		
I am most afraid of Corona	Strongly disagree	17 (20%)	40 (48%)	27 (32%)	3.16 ± 1.08	*F* = 1.109	0.331
	Disagree	14 (16%)	42 (48%)	32 (36%)	3.27 ± 0.89		
	Neutral	17 (10%)	86 (52%)	61 (37%)	3.33 ± 0.78		
	Agree	16 (14%)	51 (46%)	45 (40%)	3.43 ± 0.89		
	Strongly agree	7 (12%)	33 (56%)	19 (32%)	3.38 ± 0.9		
It makes me uncomfortable to think about Corona	Strongly disagree	16 (23%)	33 (46%)	22 (31%)	3.13 ± 1.14	*F* = 0.776	0.461
	Disagree	14 (11%)	58 (46%)	55 (43%)	3.4 ± 0.81		
	Neutral	18 (13%)	78 (55%)	45 (32%)	3.27 ± 0.85		
	Agree	20 (16%)	65 (50%)	44 (34%)	3.33 ± 0.82		
	Strongly agree	3 (8%)	18 (46%)	18 (46%)	3.53 ± 0.97		
My hands become clammy when I think about Corona	Strongly disagree	24 (18%)	65 (47%)	48 (35%)	3.27 ± 1.03	*F* = 0.565	0.568
	Disagree	17 (10%)	76 (46%)	73 (44%)	3.45 ± 0.8		
	Neutral	15 (12%)	75 (59%)	38 (30%)	3.22 ± 0.79		
	Agree	12 (22%)	25 (45%)	18 (33%)	3.26 ± 0.92		
	Strongly agree	3 (14%)	11 (52%)	7 (33%)	3.35 ± 1.1		
I am afraid of losing my life because of Corona	Strongly disagree	18 (18%)	44 (44%)	38 (38%)	3.32 ± 1.07	*F* = 1.096	0.335
	Disagree	18 (15%)	60 (50%)	41 (34%)	3.25 ± 0.84		
	Neutral	15 (12%)	72 (56%)	42 (33%)	3.27 ± 0.79		
	Agree	15 (15%)	46 (46%)	39 (39%)	3.35 ± 0.87		
	Strongly agree	5 (8%)	30 (51%)	24 (41%)	3.53 ± 0.9		
When I watch news and stories about Corona on social media. I become nervous or anxious	Strongly disagree	16 (18%)	34 (39%)	38 (43%)	3.37 ± 1.12	*F* = 1.604	0.202
	Disagree	15 (12%)	65 (50%)	49 (38%)	3.32 ± 0.85		
	Neutral	19 (13%)	78 (53%)	49 (34%)	3.3 ± 0.81		
	Agree	15 (14%)	55 (52%)	36 (34%)	3.32 ± 0.82		
	Strongly agree	6 (16%)	20 (53%)	12 (32%)	3.27 ± 1.01		
I cannot sleep because I'm worrying about getting Corona	Strongly disagree	21 (13%)	79 (48%)	64 (39%)	3.39 ± 0.98	*F* = 2.226	0.109
	Disagree	19 (12%)	67 (44%)	67 (44%)	3.4 ± 0.82		
	Neutral	14 (13%)	65 (62%)	26 (25%)	3.15 ± 0.79		
	Agree	14 (22%)	30 (47%)	20 (31%)	3.21 ± 0.89		
	Strongly agree	3 (14%)	11 (52%)	7 (33%)	3.32 ± 1.1		
My heart races or palpitates when I think about getting Corona	Strongly disagree	20 (12%)	84 (51%)	62 (37%)	3.37 ± 0.97	*F* = 0.929	0.396
	Disagree	20 (14%)	63 (43%)	63 (43%)	3.38 ± 0.81		
	Neutral	15 (13%)	69 (61%)	30 (26%)	3.17 ± 0.81		
	Agree	13 (22%)	27 (47%)	18 (31%)	3.23 ± 0.89		
	Strongly agree	3 (13%)	9 (39%)	11 (48%)	3.55 ± 1.11		

## Discussion

This study assessed the relationship between nurses' risk assessment and management, fear perception, and mental wellbeing during the COVID-19 pandemic in Saudi Arabia.

Findings in the risk assessment part 1 reported that most nurses (83.2%) provide direct patient care to patients infected with COVID-19. Previous literature showed that frontline workers, such as the HCPs who provide direct care to patients with COVID-19, are at risk of getting infected, as was the case in the study conducted in Saudi Arabia (43, 44). The results are congruent with other recent research on the high-risk perception among nurses during Saudi Arabia's COVID-19 pandemic (45).

Nurses are particularly at risk for contracting COVID-19 because they have to come in direct contact with suspected COVID-19 patients and treat multiple infections simultaneously (45). The nurses' work during this pandemic made them more vulnerable to risks such as infection. In fact, this is confirmed in the results of a phenomenological study in which the participants expressed that they were at a high risk of COVID-19 infection and that this type of vulnerability is unavoidable (46). As of November 30, 2020, the number of nurses infected with COVID-19 in the Kingdom of Saudi Arabia was already 57,159 HCPs (47). This is most likely because COVID-19 infection is higher among HCWs than in the general community, with 2747 cases per 100,000 frontline HCWs compared to 242 cases per 100,000 people in the general population (48, 49). Given the fact that nurses provide direct contact with the patient, it is indeed logical that they commonly contract the infection. This possibility could explain the increased risk of COVID-19 among the HCP, particularly the nurses.

In the Part 2 risk assessment, most of the respondents wear PPE and practice hand hygiene before and after any clean or aseptic procedure. The occurrence of pandemic affected the usage of PPE and its perceived importance by the HCPs. In a previous study, HCWs reported that PPE was frequently used and perceived as more important than the reports before the pandemic (50). Medical-ward nurses, in particular, are quite conscientious about following standard precautions like wearing masks and washing their hands before and after handling PPE (1). This might be because the HCPs recognize the additional protection in wearing PPE and practicing hand hygiene throughout this pandemic, especially for those directly caring for COVID-19 patients. The infection modes of COVID-19 were airborne, droplets, and close contact with an infected individual. Thus, wearing appropriate PPE can protect against infectious microorganisms that help in the transmission of COVID-19 infection (12). This could be why most of the nurses in this study reported wearing PPE and practicing hand hygiene before and after procedures. Despite the benefit and the additional protection, it can pose problems such as the low adherence of nurses to using PPE and not practicing hand hygiene; these problems remain a challenge for healthcare institutions. A study by Keleb et al. (51) revealed that the Northeastern Ethiopian HCP's compliance with PPE, equipment utilization, and hand hygiene was low. HCPs in the UK also reported suboptimal adherence to personal protective measures (52). The discrepancy in the results may be attributed to the difference in the setting where the nurses reportedly work. Smith et al. (54) discussed in their study that the nurses' adherence to PPE was associated with the training received by the HCWs regarding safety and health in the workplace. In Saudi Arabia, nurses reported receiving sufficient training on the preventive measures for COVID-19 (24). This could be why nurses frequently use the gear because they are aware of the protection they can get against COVID-19.

In Part 3, risk assessment, although almost all nurses are vaccinated, they are still afraid of being infected while working with a positive patient. These findings are supported by the study by Zhang et al. (53); they revealed that nurses did not change their protective behavior even after getting vaccinated. While COVID-19 vaccines are effective for immunizing people, they do not guarantee 100% complete protection (12). Even if a person is fully vaccinated, there is still a possibility that they will contract the virus. This could be why the respondents still feared being infected even after vaccination. Although this might be the case for the nurses, additional protection against COVID-19, such as getting vaccinated and adhering to protective behavior, is much better than working with no protection against the virus. Thus, it is essential to address the vaccine hesitancy of HCPs and the general population for an additional layer of protection.

Other findings of the study reported that nurses have moderate positive mental wellbeing, indicating good mental health during the pandemic. The result is congruent with the study by Abo-Ali et al. (54), in which the majority of the HCPs in Saudi Arabia had positive mental wellbeing scores during the COVID-19 pandemic. One of the probable reasons nurses felt positive mental wellbeing despite the COVID-19 pandemic is that the situation as of 2022, when this study was conducted, is under control. As of June 2022, over 71.5% of people in Saudi Arabia are vaccinated, and the newly reported cases of COVID-19 in the last seven days were only 3,830, which is lower than at the onset of the pandemic (55). This indicates that COVID-19 cases relatively decreased, which made the nurses' work more relaxed. Furthermore, nurses might have already been used to the protocol and the procedures that are necessary to perform for COVID patients, which allowed them to work more smoothly. Therefore, regular drills and training for disaster management are suggested to help HCWs adjust and use the routine.

The current study found that nurses reported a low fear of COVID-19 scores. This level of fear is lower compared to the study of Mohsin et al. (56), which revealed that Saudi nurses have an overall low or moderate level of fear of COVID-19. The low fear of nurses toward COVID-19 may be attributed to the policies that were clearly established to mitigate the negative situation brought on by the pandemic. For instance, aside from the vaccine rollouts in Saudi Arabia, the Ministry of Health developed COVID-19 guidelines based on recent COVID-19 research to help HCPs deal with COVID-19 patients. The MOH protocol for patients who tested or were thought to be positive for COVID-19, airway management, and mechanical ventilation protocols was detailed in instruction manuals (57). Nurses, as human beings, fear the unknown. However, due to the continuous publication of guidelines and materials to guide the HCPs, the facts and other important information became known, which might be why they are no longer afraid of dealing with it. Considering this, healthcare institutions may review their COVID-19 policies and protocols to clarify implementation.

There is a significant association between some demographic profile variables and perceived risk assessment of nurses' mental wellbeing scores. Specifically, nurses' age is significantly associated with mental wellbeing. It implies that the older the nurses are, the better their mental wellbeing. This result agrees with the results in a study by Cheung and Yip (58), which revealed that age and years of experience negatively correlate with poor mental health. Specifically, younger nurses with less experience had lower mental health. This is worth noting since older nurses might have acquired more extensive experience in nursing practice, which allowed them to adjust well to the situation and become well-versed with the routine and procedures. Although some studies identified specific age brackets of nurses with better mental wellbeing, there is no consensus among the researchers regarding the specific age of nurses with better mental wellbeing. The only common denominator in their findings was that they all agreed on the result that older nurses have better mental wellbeing. Healthcare institutions may help younger nurses to achieve stable and better mental wellbeing by providing opportunities for them to acquire more experience through various training programs and life coaching to support their journey and help them to manage their time while balancing their career and family life.

The nationality of nurses is associated with mental wellbeing scores. Specifically, non-Saudi nurses have higher perceived mental-wellbeing scores than Saudi nurses. This finding is backed by recent research (7), which revealed that nationality was a significant predictor of nurses' psychological burden. In their study, Filipino and Indian nurses achieved lower mean scores for depression, anxiety, and stress than Saudi nurses. This may be attributed to the support system that non-Saudi nurses received from their families and friends. As expatriate nurses in Saudi Arabia, Filipino nurses were known to have close familial ties (59). Consistent and close communication with families might be one of the reasons why non-Saudi nurses are provided with adequate social support. Having social support from friends had an indirect effect on the resilience of the nurses (59). This might be the reason why better mental wellbeing is observed among expatriates. With this in mind, it is recommended that healthcare institutions provide a good support system for all the HCPs to rely on amidst the pandemic.

Next, the region where nurses currently work has been significantly associated with mental wellbeing. This is worth noting since different regions might have different hospital policies, human resource management policies, providing care policies, administrative policies, and a diversity of healthcare professionals' compliance with several policies and procedures of each organization. Considering the complex nature of the healthcare industry's structure, this may affect their mental wellbeing. According to WHO (1), workplace environments also have different health and safety issues, communication, management practices, employee support, and organizational tasks and objectives, all affecting their mental health. Although the current study included the area of practice, findings have shown that it has no significant association with the perceived risk in relation to their mental wellbeing. Nonetheless, it negates the study by Cruz et al. (17), in which they discussed that emergency nurses have a higher infection risk, higher workload, fatigue, and helplessness, which could be associated with a high depression level in ED nurses. A recent study in China conducted by Chen et al. (60) revealed that ICU nurses and those assigned to COVID-19-designated hospitals and departments have a higher developing symptoms risk, the same with posttraumatic stress disorder (PSTD). This could indicate that the workplace where nurses are assigned might have brought risks that could affect their mental wellbeing. However, the method did not include risk factors for mental health that may be present in the working environment. Thus, further research determining risk factors for mental health and relationships between work environments is warranted.

Finally, nurses' risk assessment is associated with mental wellbeing scores. This result agrees with the results of the previous studies that the risks encountered by the nurses as frontline workers during the COVID-19 pandemic increase their mental health issues (13, 14). This is worth noting since nurses, as health care professionals, render direct care to patients with COVID-19, which indicates that they are more exposed to the risks affecting their mental wellbeing. A study showed that HCPs working in direct contact with patients who tested positive for COVID-19 experienced two times the risk of depression and anxiety compared to HCWs with less exposure to COVID-19 (61). Since the newly reported cases are constantly decreasing, the possibility of the risks that nurses might experience decreases as well, thereby maintaining their mental health. Continuous support on vaccine rollouts, adherence to wearing protective gear, and hand hygiene behaviors among frontline workers may enable them to fully control adverse events that currently occur during the pandemic.

## Conclusion

The findings indicate that the risks posed by the COVID-19 pandemic affect nurses' mental wellbeing. Due to their frequent and close interaction with patients with COVID-19, nurses are at an increased risk for mental health issues. The results revealed that most nurses provide direct patient care to patients with COVID-19, state the importance of wearing PPE, and perform hand hygiene before and after any clean or aseptic procedure. Meanwhile, although almost all nurses are vaccinated, they are still afraid of being infected with COVID-19 while working with a patient tested positive for COVID-19 and reported low fear of COVID-19 scores. Additionally, the results reported that the older the nurses, the better their mental wellbeing scores; non-Saudi nurses have higher perceived mental wellbeing scores than Saudi nurses, and different working environments correspond to different mental wellbeing scores. Finally, nurses' risk assessment is associated with mental wellbeing scores. Therefore, the study's findings proved that despite getting vaccinated and the nurses' adherence to hand hygiene behaviors, they are still susceptible to fear of COVID-19, which will impact their mental wellbeing.

## Limitations of the study

Some limitations should be considered in the findings. This study used a cross-sectional design with online survey questionnaires. Hence, causality cannot be conclusively inferred. Also, convenient sampling was used, which led to an inability to generalize the findings. The study was conducted after the COVID-19 vaccine was introduced and after 2 years of pandemic experience. Further interventional studies' management assessment of nurses' mental wellbeing and various coping mechanisms for nurses' mental health during and after the pandemic are warranted.

## Implications

These findings may help us understand the implications of healthcare institutions' policies, which are relevant to improving healthcare workers' mental health during the COVID-19 pandemic. The result of the study could provide information as a benchmark for promoting positive mental health while reducing the risk and fear perception of COVID-19. The results improved our understanding of the links between the described variables and nurses' mental wellbeing, contributing to effective prevention programs and therapeutic intervention development methods. At a nursing practice level, the results may be useful for nursing management in identifying and supporting nurses' COVID-19 anxiety levels and risk perception to mitigate risks. Interventions focused on improving mental wellbeing coping strategies might increase nurses' confidence and resilience in the face of unpredictable adverse events on psychological wellbeing that seek to reduce the anxiety and fear induced by the pandemic to promote both the implementation of preventive behaviors against COVID-19 and wellbeing in the evaluated Latin American and Caribbean countries. The authorities in the healthcare sector must first recognize that nurses are most susceptible to poor mental health during the COVID-19 pandemic. Creating an awareness program and life coaching may help nursing professionals to be mindful of their mental health during the pandemic. A comprehensive review of the local COVID-19 policies and protocol may be essential to ensure it is implemented. Enabling support systems for nursing professionals is also important to address adequate support for the protective measures against COVID-19. They must be physically and mentally supported to maintain healthy mental wellbeing during the COVID-19 pandemic.

## Data availability statement

The raw data supporting the conclusions of this article will be made available by the authors, without undue reservation.

## Ethics statement

The studies involving human participants were reviewed and approved by Institutional Review Board—Hafr Albatin committee (KACST No. H-05-FT-083). The patients/participants provided their written informed consent to participate in this study.

## Author contributions

RA-D, SA, MB, MA, HA, KA, MSA, BA, RF, NA, KA, JA, and JUA contributed from the data collection, data analysis and interpretation, drafting of the article, and critical revision of the article. All authors contributed to the article and approved the submitted version.

## Conflict of interest

The authors declare that the research was conducted in the absence of any commercial or financial relationships that could be construed as a potential conflict of interest.

## Publisher's note

All claims expressed in this article are solely those of the authors and do not necessarily represent those of their affiliated organizations, or those of the publisher, the editors and the reviewers. Any product that may be evaluated in this article, or claim that may be made by its manufacturer, is not guaranteed or endorsed by the publisher.
